# Elements of Zoology; or Lectures on the Anatomy, Physiology, Classification, and Habits of Animals

**Published:** 1838-01

**Authors:** 


### Art. XIX.

*Elémens de Zoologie; ou Leçons sur l'Anatomie, la Physiologie, la Classification, et les Mœurs des Animaux.* By M. H. Milne-Edwards, d.m., &c.

*Elements of Zoology; or Lectures on the Anatomy, Physiology, Classification, and Habits of Animals.* By Dr. H. Milne-Edwards.—*Paris*, 1834-7. 8vo. pp. 1066.

The recent appearance of the last part of this valuable work leads us to take the opportunity of bringing it before our readers, and strongly urging it upon their attention. Its author must be known to all of them as a countryman of our own, and as one of the most distinguished naturalists of the present day. Though professedly an elementary work, it is easy to trace in it the pen of a master in the science; and we know of no book which we can with more confidence recommend to those of our younger friends who are desirous of acquiring such a knowledge of zoology as may aid them in more profound and scientific researches in Comparative Anatomy and Physiology. It is not the intention of the writer to detail the minutiæ of genera and species, but rather to point out the peculiarities of structure which characterize each principal subdivision of the animal kingdom; and this is done in a manner at once clear, concise, and attractive. The anatomical and zoological descriptions are illustrated by more than five hundred woodcuts, many of which will bear a comparison with the best that have been recently produced in this country; and the cheapness of the volume is by no means its least recommendation. We shall be very glad to see the general diffusion of works like this lead to an increased attention to natural history among the members of our profession in this country; and we are persuaded that the student who devotes to such rational employment a portion of that time which is generally given to idle and frivolous amusement, will find no reason to regret his choice in after life, nor will he usually experience any difficulty in combining a moderate share of such pursuits with the faithful and zealous discharge of his more strictly professional duties.

